# Is Telomere Length Optimized in Hatchling Sand Lizards?

**DOI:** 10.1111/ede.70020

**Published:** 2025-11-04

**Authors:** Mats Olsson, Emily Miller, Nicky Rollings, Erik Wapstra, Richard Shine

**Affiliations:** ^1^ Department of Biological and Environmental Sciences University of Gothenburg Gothenburg Sweden; ^2^ School of Biological Sciences, University of Wollongong, Northfields Avenue Wollongong New South Wales Australia; ^3^ Office of the Deputy Vice‐Chancellor (Research), The University of Sydney Sydney New South Wales Australia; ^4^ School of Life and Environmental Sciences, The University of Sydney Sydney New South Wales Australia; ^5^ School of Natural Sciences, University of Tasmania Hobart Tasmania Australia; ^6^ School of Natural Sciences, Macquarie University Sydney New South Wales Australia

**Keywords:** adaptation, hatchling telomere length, Lacertidae, life span, lifetime reproductive success, offspring recruitment, Squamata, telomere elongation

## Abstract

Telomeres (repeat‐DNA‐protein structures primarily located at the ends of chromosomes) protect coding DNA against attacks by reactive molecules and the cells’ own DNA repair systems. If that capacity is costly, but enhances an individual's viability, we might expect to see natural selection acting on telomere length: that is, individuals with optimal telomere lengths should have higher lifetime reproductive success than conspecifics with shorter or longer telomeres. Some recent studies on humans broadly support that prediction, but no data are available for free‐ranging ectothermic vertebrates that, unlike mammals, can facultatively adjust telomere length during an individual's lifetime. In our decade‐long study of a natural population of sand lizards (*Lacerta agilis*), including measurement of 2736 telomeres across > 1700 hatchling lizards and their > 500 parents, but with a very high hatchling mortality reducing later‐life sample sizes, we found that lifespan, lifetime reproductive success and offspring recruitment rate were highest for hatchlings with “average‐length” telomeres. Hatchlings with shorter‐than‐average telomeres elongated their telomeres during juvenile life, attaining the population‐average telomere length by the time of sexual maturity; but that compensatory telomere growth was associated with lower body condition.

## Summary

Our manuscript is based on a long and detailed ecological study of free‐ranging sand lizards, incorporating data on hatchlings’ future lifespan, lifetime reproductive success and recruitment of breeders from their produced young. We use these unique data to explore a topic that has been ignored in previous work—that is, how does natural selection in an ectothermic (“cold‐blooded”) species act on a high‐profile trait (telomere length) that has attracted massive research effort in endothermic (“warm‐blooded”) vertebrates? Our analyses support the hypothesis that telomere length in hatchlings is under selection in these free‐ranging sand lizards, with benefits high enough to warrant costly adjustment processes during juvenile life.

## Graphical abstract

The graphical abstract image depicts the complex interplay of factors driving telomere dynamics (with permission from Dr. Chris Friesen).

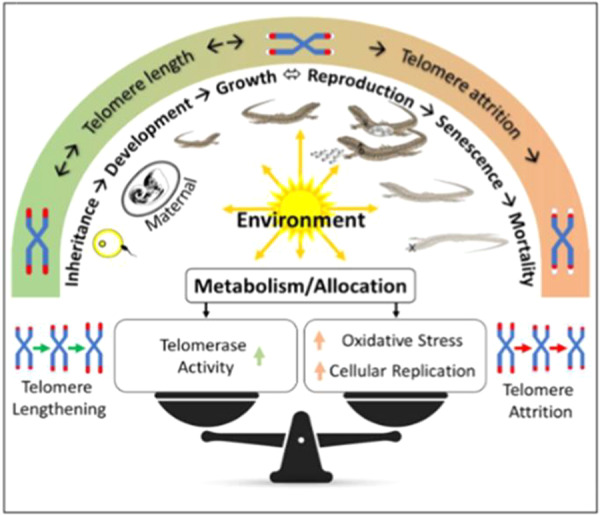

## 1 Introduction

Many physiological and genetic processes are highly conserved among living organisms but exhibit lineage‐specific divergences in major aspects. As a result, studies on functionally similar systems in disparate species provide an opportunity to see such systems in a broader context. For example, we now have extensive details on physiological mechanisms and processes in humans but we need the evolutionary background of those traits in other species to appreciate our evolutionary history. For vertebrates, one of the most exciting opportunities comes from comparisons between endothermic (“warm‐blooded”) and ectothermic (“cold‐blooded”) taxa. The evolution of constant‐temperature, energy‐expensive, high‐metabolic‐rate physiologies in mammals and birds has been accompanied by a loss of facultative variation in many characteristics. Thus, for example, traits ranging from metabolic rates to sex‐determining systems exhibit less thermal sensitivity in endotherms than in ectotherms (Agata and Nomura 2024; Gillooly et al. 2017; Xiong et al. 2024).

In the current paper, we explore a fundamental aspect of chromosome morphology that is common to all vertebrates but for which control systems differ profoundly between endothermic and ectothermic species. Telomeres are DNA‐protein ‘endcaps’ of chromosomes that protect against an organism's own DNA repair‐systems and against other challenges such as free radicals. They also modify gene expression and contribute to genomic integrity (Monaghan and Haussmann 2006). Most of our understanding of telomere biology comes from studies of endotherms, in particular humans and laboratory mice (Blasco 2003; Chan and Blackburn 2004). For a broader understanding, however, we need to look at evolutionary processes in free‐ranging organisms. Ectotherms are of special interest in this regard; not only do they make up some 99% of all living species, but many of those ectotherms possess a capacity that is generally lacking in endotherms: the ability of adults to produce an enzyme (telomerase) that enables facultative adjustment of telomere length (TL) in somatic tissues (Olsson et al. 2018). This is a major underlying reason why telomeres are maintained or elongated during an individual's lifetime in many ectotherms including Atlantic salmon (*Salmo salar*) (McLennan et al. 2018), cave‐dwelling amphibians (*Proteus anguinus*) (Voituron et al. 2023), leatherback turtles (*Dermochelys coriacea*) (Plot et al. 2012), sand lizards (Olsson et al. 2024), common lizards (*Lacerta vivipara*) (McLennan et al. 2019), highland populations of ocellated snow skinks (*Niveoscincus ocellatus*) (Fitzpatrick et al. 2021), and tropical pythons (*Liasis fuscus*) (Ujvari and Madsen 2009). In tropical frill‐necked lizards (*Chlamydosaurus kingii*) telomeres elongate until middle age and then decline (Ujvari et al. 2017). In addition, in termite castes (*Lasius niger*) of extremely different lifespans (e.g., 2–3 months vs. 28 years), more long‐lived castes have longer telomeres (Jemielity et al. 2007). In contrast, the length of an endotherm's telomeres typically decreases during its life (Glousker et al. 2020; Jia et al. 2015; Lanna et al. 2022; Mannherz and Agarwal 2023; Ujvari et al. 2022; Vertecchi et al. 2022; Zade and Khattar 2023) with a net attrition rate set by nucleotide loss when cells divide (i.e., the end‐replication problem; Monaghan 2024) and when exposed to eroding molecules, such as free radicals (Reichert and Stier 2017).

Endotherms do not entirely lack the ability to facultatively adjust TLs, but that ability is less marked than in ectotherms (Barrett and Richardson 2011; Chik et al. 2022; Eastwood et al. 2023; Kärkkäinen et al. 2022; Reichert and Stier 2017; Remot et al. 2020; Salmón and Burraco 2022; Tobler et al. 2022; Wilbourn et al. 2018). As a result, low variance in rates of telomere attrition and elongation among individuals within a population of endotherms renders it difficult to measure correlates of such variation—the data needed to answer the fundamental question of how TL affects components of Darwinian fitness such as lifespan and lifetime reproductive success (LRS). Because somatic telomerase production is widespread in ectotherms, however, we can potentially measure links between telomere dynamics (rates of repair and elongation) and components of fitness (Olsson et al. 2011).

Although human telomeres have been a subject of detailed research since the 1960s (Baird 2008; Epel et al. 2004; Hertzog 2006; Lansdorp 2022a; Lansdorp 2022b; Monaghan and Haussmann 2006), studies on non‐human (and especially, free‐ranging rather than laboratory) populations have been less numerous. Nonetheless, such studies have included work on correlations between telomeres and disease (Ujvari et al. 2022), in‐/outbreeding (Olsson et al. 2022; Pepke et al. 2022), seasonality (Axelsson et al. 2020a; Hoelzl et al. 2016), social environment(Burraco et al. 2022; Epel et al. 2004), and components of fitness such as ageing (Bénard and Hekimi 2002; Heidinger et al. 2016), senescence(Bénard and Hekimi 2002), and LRS (Eastwood et al. 2023; Olsson et al. 2011). To our knowledge, though, there have been no studies to date of the relationship between TL at hatching, and its dynamics, and individual variation in Darwinian fitness traits in an ectotherm.

**Figure 1:**
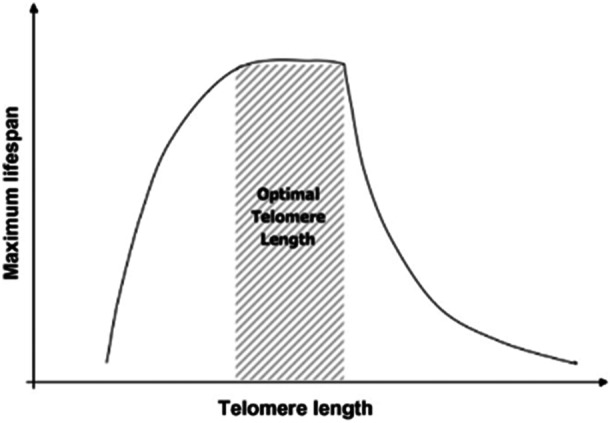
Re‐worked Figure 2 from Kappei and Londoño‐Vallejo publication depicting a scenario in humans when telomere length is favored by [ageing] selection up to a stage when costs from disease [largely cancer] are expected to counter further positive effects from TL. The authors concluded: “too short telomeres lead to premature ageing and increased risk of ageing‐assisted diseases…and too long telomeres may have negative impacts through an increased risk of proliferative‐related disorders.” Our current work on hatchling sand lizards largely concurs with their predictions even though underlying cost mechanisms for telomere dynamics may differ in a species with somatic telomerase. *Source:* Figure 1 is reproduced with the permission of Dr. Dennis Kappei.

What kind of relationship might we expect between telomere traits and fitness measures in ectotherms? Given the available information from endotherms, several authors have suggested that TL in humans may be under stabilizing selection, with lower fitness accruing to shorter‐than‐usual or longer‐than‐usual telomeres. For example, in an influential paper in 2008, Dennis Kappei and Arturo Londoño‐Vallejo (Kappei and Londoño‐Vallejo 2008) concluded that there may be an “optimal telomere length” (their Figure 2. p. 23, here reworked with the authors’ permission in Figure 1). That is, “too short telomeres lead to premature ageing and increased risk of ageing‐assisted diseases…and too long telomeres may have negative impacts through an increased risk of proliferative‐related disorders” (Kappei and Londoño‐Vallejo 2008). Consistent with that hypothesis, Stone et al. (2016) found that alleles associated with short telomeres increase the risk for atherosclerosis in humans, whereas alleles associated with long telomeres increase the risk of cancer and reduce the risk of atherosclerosis (but see also Aubert et al. 2012). Stabilizing selection also predicts relatively lower variance around the mean in a trait, relative to a trait that has been less exposed to such variance‐reducing forces. This pattern is what we found in a previous analysis contrasting hatchling and adult TLs in sand lizards, with considerably more variance in adult TL (Olsson et al. 2024; Figure 4), and encouraging the work reported on here.

We used our long‐term data set on sand lizards to test the following predictions with reference to telomere optimization: if TL at hatching is under stabilizing selection, individuals with close‐to‐population‐average TLs are expected to exhibit higher‐than‐average (i) lifespan; (ii) LRS; and (iii) offspring recruitment rate to the next generation. Also, because lizards are capable of repairing telomeres during their lifetime, we expect that (iv) each individual will adjust TL in relation to costs for this and available resources (e.g., nucleosides), and that (v) because TL repair and maintenance is metabolically expensive in this system (Olsson et al. 2024), individuals that facultatively adjust their TL during juvenile life will pay a cost in terms of energy balance (as measured by body condition). Such a cost might help to explain why the ability to facultatively adjust TL during ontogeny has been lost in most endotherms where metabolic costs are higher.

## 2 Materials and Methods

### 2.1 Model System

Sand lizards (*Lacerta agilis*) are small (up to 20 g), sexually dimorphic (females larger), sexually dichromatic ground‐dwellers (males green, female brownish‐gray) with one of the largest distributions of any reptile, ca. 8000 × 5000 km with their main abundance in central Europe. Our study population (Asketunnan; N570 22′ E110 58′) is situated ca. 50 km south of Gothenburg on the Swedish west coast and has been studied with respect to evolutionary ecology and genetics since 1984; the data in the current manuscript were gathered from 1998 to 2008. Lizards were monitored from mid‐April at emergence from hibernation until the end of the mating season (ca. mid‐June) on all days with suitable weather (heliothermic ectotherms depend critically on favorable basking conditions). Each lizard was captured by noose or by hand, given a unique claw/toeclip code, and marked with a numbered tape tag on its back (renewed after skin‐shedding) to facilitate monitoring and studies in behavioral ecology and genetics (e.g., Anderholm et al. 2004; Olsson 1993, 1994; Olsson et al. 2003). Over the decade‐long study we made 3688 observations of 576 individually marked males, and 2288 observations of 528 females and their 4534 offspring. In those offspring, we measured TLs 1854 times on 1840 hatchlings. Because of the very high mortality on these ca. 1 g hatchling lizards (ca. 90% mortality depending on year (Olsson et al. 2022; Olsson and Madsen 2001), our sample sizes dropped to 112 on individuals for which we managed to acquire lifetime fitness data such as LRS (for some with repeat telomere observations as adults, resulting in 184 telomere observations for these 112 hatchlings).

Our analyses do not represent selection coefficients in the strict quantitative genetic sense, because the high mortality rates of these small hatchling lizards include individuals that have died for reasons other than telomere‐related factors, probably often random, that would complicate the analyses and their interpretations of links between telomeres and relative fitness effects. To increase statistical power, we used maximum sample sizes for the different analyses presented in this manuscript, which therefore vary to some extent depending on available data.

Previous work shows that maturation time is ca. 2–3 years (Olsson and Shine 1996), maximum lifespan in our population is ca. 10 years (Olsson and Shine 1996) and that females live on average 3.7 ± 0.18 years and males 4.11 ± 0.23 years (current study). The mating system is highly promiscuous with some 70% of clutches sired by multiple males (Olsson et al. 2005; Olsson et al. 2011; Olsson et al. 1996; Olsson et al. 2011) with a bias in probability of paternity towards the least related male in situations of sperm competition and cryptic female choice (Olsson et al. 1996). TL in males competing for fertilization in staged mating trials predicts probability of paternity (Pauliny et al. 2018); this agrees with results from human IVF technology showing a positive relationship between sperm telomeres and sperm viability and swimming capacity (Santiso et al. 2010) (but see also lack of such relationships in bovines: (Ribas‐Maynou et al. 2022). Furthermore, male telomere elongation capacity in adult sand lizards is a significant predictor of male LRS, suggesting that resource‐driven telomere maintenance late in life is linked to male reproductive success (Olsson et al. 2024).

Female sand lizards lay a single annual clutch of 5–15 eggs, depending on maternal body size. Approximately 1 week before egg‐laying, when females show egg contours along their abdomens, gravid females were brought to facilities at the University of Gothenburg, Sweden. The lizards were weighed to the nearest 0.001 g, measured snout‐to‐vent (SVL) to the nearest mm, and had a condition index calculated as a residual from a mass‐SVL regression (Olsson 1994; Olsson and Shine 1997). The lizards were marked by claw‐clipping for identification (Olsson 1994), and kept individually in cages (40 × 50 × 60 cm) containing a flat rock on wet sand for egg‐laying, and a 40‐W spotlight at one end to allow thermoregulation. Eggs were collected within hours of laying and incubated at 25°C, a temperature which optimizes hatching success and minimizes developmental asymmetries in this species (Zakharov 1989). All clutches were incubated individually in separate boxes, all in the same incubator, with eggs hatching after approximately 40 days. Hatchlings were released within days of hatching (only awaiting ideal release weather for ectotherms) at locations identified as benign in terms of survival (sand lizards are IUCN red‐listed in Sweden) but at random with respect to juvenile identity at the Asketunnan study site. A corridor of ca. 600 m around the circumference of the study site was monitored routinely to control for dispersal effects as a confounder of mortality and lifespan. Because the maximum recorded dispersal distance was 135 m, the 600 m corridor ensures accurate estimation of mortality and lifespan (57–59).

### 2.2 Genotyping and Parentage Assignment

DNA was extracted from blood of 4534 individual lizards for parentage assignment (including adults, juveniles, plus tissue samples from embryos that died during incubation and were not used for telomere assessment in the current study). Parentage was assigned using 17–21 microsatellite loci (Olsson et al. 2011). From the 3627 eggs that hatched, a total of 2601 juveniles were successfully assigned a father (Olsson et al. 2011). All blood samples were stored at negative 80°C from sampling to labaratory procedures, and all DNA was stored at negative 80°C subsequent to extraction. LRS was calculated as an individuals’ cumulative number of offspring sired through life as assigned with molecular genotyping (Olsson et al. 2011). Recruitment of offspring to the breeding population was assessed by longitudinal monitoring of parentage‐assigned offspring.

### 2.3 TL Measurements: qPCR

In 2011–2014 DNA was extracted from whole blood using the Qiagen Puregene blood kit (Cat No. 158467). Reptiles have nucleated erythrocytes (red blood cells) that make up ca. 95% of all blood cells (Glenn and Armstrong 2019) and, hence, is the overwhelmingly dominant telomeric DNA source. Sand lizards do not have interstitial telomeres (Matsubara et al. 2015; Rovatsos et al. 2015) and we therefore measured TL using quantitative polymerase chain reaction (qPCR) (Nussey et al. 2014). All qPCR work followed Cawthon (2002) and Criscuolo et al. (2009) with slight modifications (Axelsson et al. 2020b). To evaluate DNA concentration and purity, we used a PHERAstar F5 Spectrophotometer (BMG Labtech, Germany). The total yield averaged 527 ng/µL per sample, with high molecular purity (mean A260/280 = 1.76; *n* = 2476). Samples with low yield and/or low quality were excluded, and other samples were diluted to a working concentration of 20 ng/µL. The limit for exclusion was set to 5 ng/µL and 1.4 for all our analyses but these applied only to juvenile samples. DNA concentrations ranged from 5.96 to 1090.61 ng/µL. All samples were diluted to 20 ng/µL, and nine samples that were less than 20 ng/µL were not diluted and the number of microliters of DNA added was increased to a total of approximately 20 ng/µL. The A260/280 range was 1.4–1.95.

Previously published primers (Criscuolo et al. 2009) were used to amplify the reference gene glyceraldehydes‐3‐phosphate dehydrogenase (GAPDH) and telomere primers (Tel1b and Tel2b). A standard curve was generated for GAPDH and telomere primers using six fivefold serial dilutions of DNA from an arbitrary *L. agilis* sample to generate a reference curve. Each run included a positive control, no template control (NTC) and the same reference standard to control for the qPCR's amplification efficiency and set up to the threshold Ct value; every sample was run in triplicate. For maximized precision, if a triplicate had a standard deviation > 0.3 it was rerun. The DNA concentrations at each point of the standard curve were: (1) 500 ng (log template DNA = 2.7), (2) 100 ng (log template DNA = 2.0), (3) 20 ng (log template DNA = 1.3, (4) 4 ng (log template DNA = 0.6), (5) 0.8 ng (log template DNA = −0.1), (6) 0.16 ng (log template DNA = −0.8).

In 2022, we outsourced an analysis for another telomere project (Olsson et al. 2024) to the TATAA molecular genetics consultants in Gothenburg, Sweden, in which we took the opportunity to also analyse ValidPrime (VP) Vertebrate assay (#A108S25, TATAA Biocenter) (Laurell et al. 2012) to look for congruence in TL T/S ratio estimation using GAPDH and Valdiprime data as reference genes for our calculations. Our motivation for this procedure was that ValidPrime is highly optimized and specific to a non‐transcribed locus of gDNA that is present in exactly one copy per haploid normal genome (Laurell et al. 2012). The efficiency of each primer pair was tested by running a 7‐point standard curve with four replicates for each point. The dilution series comprised fourfold dilution steps while template concentrations ranged from 25 ng/reaction to 0.00625 ng/reaction. If the standard deviation of the replicates were above 0.5 the reactions were re‐run. The template for the standard curves was obtained from a pool of extracted gDNA. The concentration of the gDNA pool was determined with spectrophotometry (Lunatic, Unchained Labs) and sequentially diluted to the required concentrations. The NTC reactions were run with four replicates to check for contamination and evaluate primer dimerization. The performance of all three primers was good with efficiencies between 87% and 93% and *R* ^2^ values ranging from 0.995 to 0.998. TLs estimated using TS ratios (Cawthon 2002) based on the telomere primers and Validprime (0.97 ± 0.63, mean E^ΔCq^ ± std) vs. telomere primers and GAPDH (0.85 ± 0.51, mean E^ΔCq^ ± std) showed very high congruence (Spearman correlation coefficient, *r*_*s*_ = 0.943, *N* = 152, *p* < 0.0001).

All our 2011–2014 PCR analyses were performed on a RotorGene6000 (Qiagen, Australia); this machine does not use plates but instead analyses samples loaded onto a circulating disk, which avoids many of the plate effects identified for other cyclers. The unique centrifugal rotary design of the Rotor‐Gene is claimed to make it the most precise and versatile real‐time PCR cycler currently available (see further at https://www.qiagen.com/us/products/discovery-and-translational-research/epigenetics/dna-methylation/methylation-specific-pcr/rotor-gene-q). Each tube spins in a chamber of moving air, keeping all samples at precisely the same temperature during rapid thermal cycling. An analysis showed that RotorGene “run number” (instead of plate number as per qPCR analyses with conventional methods) had no significant effect on our main trait of interest, deviation from the telomere mean length at hatching (*F*_1, 110_ = 0.18, *p* = 0.672). Thus, including run effects in the modeling would risk introducing spurious effects.

Using the product‐recommended PCR conditions, 11.25 µL SensiMix SYBR no‐ROX Master Mix (Bioline, Australia) was included in the 20 µL final volume of both 200 nM concentration primer sets. A total of 1 µL DNA per well was added at a concentration of 20 ng/µL, except for 9 samples that yielded less than 20 ng/µL; 2–4 µL was added for each of those latter samples. Non‐specific products were not amplified, indicated by a single peak in the melt curve analysis for each reaction. The cycle at which the fluorescence level crosses the threshold, which is proportional to the quantity of DNA in a sample, is represented by the threshold cycle values (C_t_). For each sample, C_t_ values were obtained for both genes. Each sample's TL (T) was expressed relative to the single gene control (S; GAPDH). The Pfaffl method (Pfaffl 2001) was used to calculate relative TL (T/S ratio) as the deviation in qPCR reading from a reference DNA sample (GAPDH), i.e., relative telomere to single copy (T/S) ratio (adapted from Cawthon 2002). Inter‐assay coefficient of variation (mean ± STD) for qPCR runs for telomere (*n* = 123) and GAPDH (*n* = 176) amplification were 3.85 ± 0.59% and 7.73 ± 1.87%, respectively. Mean amplification efficiency across all qPCR runs (*n* = 299) was 1.999 ± 0.013 (STD). Raw data were also double‐checked for baseline fluorescence, individual efficiencies, and window of linearity per amplicon using LinRegPCR 12.18 (Ruijter et al. 2009; Tuomi et al. 2010).

### 2.4 Statistical Methods

All analyses were performed with SAS Institute Software, Version 9.4, Carey, NC, USA. Proc MIXED was used for mixed model analyses with continuous responses and Proc GLIMMIX and Proc COUNTREG for mixed model logistic or Poisson regression models with categorical or counts responses. TLs were log transformed to meet test assumptions. Descriptive changes from hatchlings to our first observation as adults were tested with *F*‐test, supported by Levene's tests for variances. Sample sizes are analysis‐specific depending on available number of samples and reported on in association with each relevant analysis. For detailed estimation of parental effects and intraclass correlation coefficients for hatchling TLs on a larger data set without lifetime fitness data, see (30).

Telomere “attrition” is a term largely derived from studies of endotherms which generally show telomere shortening through life, an estimate that requires statistical re‐sampling parameters in the population to be controlled for (i.e., the effect that more deviant observations at first sampling will have a higher probability to end up with a second observation closer to the mean at resampling (Sudyka et al. 2019)). We therefore retained “attrition” as a general concept, even though “elongation” may be more important in ectotherms with somatic telomerase (i.e., elongation is the “negative attrition” in TL at hatchling‐minus‐adult TL subtraction). We defined “attrition” as follows (following Sudyka et al. 2019), regardless of whether this resulted in real net “attrition” (i.e., telomere shortening) or elongation (i.e., mathematically, being a negative attrition), and took regression to the mean into account by adjusting telomere dynamics using the following calculation:

$$D=p\left(X_{1}–X_{1}\text{mean}\right)–\left(X_{2}–X_{2}\text{mean}\right),$$

where p = (2rs_1_s_2_)/(s_1_^2^ + s_2_^2^),

and X_1_ = initial telomere measure, X_2_ = last telomere measure, r = correlation between X_1_ and X_2,_ and s = their standard deviation and s^2^ = their variance.

In our telomere dynamics analyses we used individual ID as a random factor when we had repeat observations for the same individual. Error of measurements could be influenced by the quality of the DNA in our telomere and GAPDH analyses and we therefore tested whether year of sampling (and hence storage time until analysis) would impact our DNA quality estimate but this was confirmed as non‐significant (effect of year, *F*_6, 159_ = 1,41, *p* = 0.213). In no case did DNA quality affect the qualitative outcome of our analyses (see test statistics in the table headings).

Lande and Arnold (Lande and Arnold 1983, p. 1218, 3rd para) warned that linear and quadratic terms may be correlated in quantitative genetic selection analyses and estimates of regression coefficients (*β*) depend on whether or not the quadratic terms are included in the regression. Here, we do not estimate quantitative genetics coefficients but use corresponding regression analyses to assess the links between telomere traits and components of fitness. Because TL at hatching and their absolute deviation from the mean were correlated, we calculated their regression coefficients in a model with both traits present but also complemented these analyses with independent significance tests for each trait. Those traits were log hatchling TL and log absolute deviation from the mean as predictors of lifespan (in years), LRS (their cumulated number of molecularly assigned offspring), and their recruited number of offspring surviving to breed in the next generation.

## 3 Results

### 3.1 Sex‐Specific TL at Hatching

A first descriptive analysis showed that females hatch with slightly shorter telomeres than do males in the Asketunnan population of sand lizards (untransformed TSratio data: males: 0.92 ± 0.003, means ± SE, *N* = 827; females: 0.89 ± 0.003, means ± SE, *N* = 868; Table 1). This result held true also when maternal ID and birth year were used as random effects (Table 1). We therefore included sex in the initial analyses below but removed sex from final analyses when not significant.

**Table 1:** Analysis of sex‐specific telomere length at hatching in Proc Mixed, SAS 9.4. SAS fixes one sex at zero against which the effect of the other sex is contrasted. The degrees of freedom were calculated using Kenward‐Rogers estimation. Birth year and maternal identity were used as random factors (significant log likelihood ratio tests for each factor, *χ* ^2^ = 555.9, *p* < 0.001 and *χ* ^2^ = 463.2, *p* < 0.001, respectively). DNA quality was a non‐significant covariate (*F* = 0.26, *p* = 0.612) and was backwards‐eliminated.

Response variable: Log telomere length at hatching						
Effect	Sex	Estimate	Standard error	DF	*t* Value	Pr > |*t*|
Intercept		0.012	0.023	7.15	0.53	0.612
Sex	F	−0.03	0.003	1616	−10.55	< 0.0001
Sex	M	0….				

Type 3 tests of fixed effects				
Effect	Num DF	Den DF	*F* Value	Pr > *F*
Sex	1	1616	111.36	< 0.0001

### 3.2 Effects of Deviation From Mean Hatchling TL (Stabilizing Selection) on Components of Fitness

**Figure 2:**
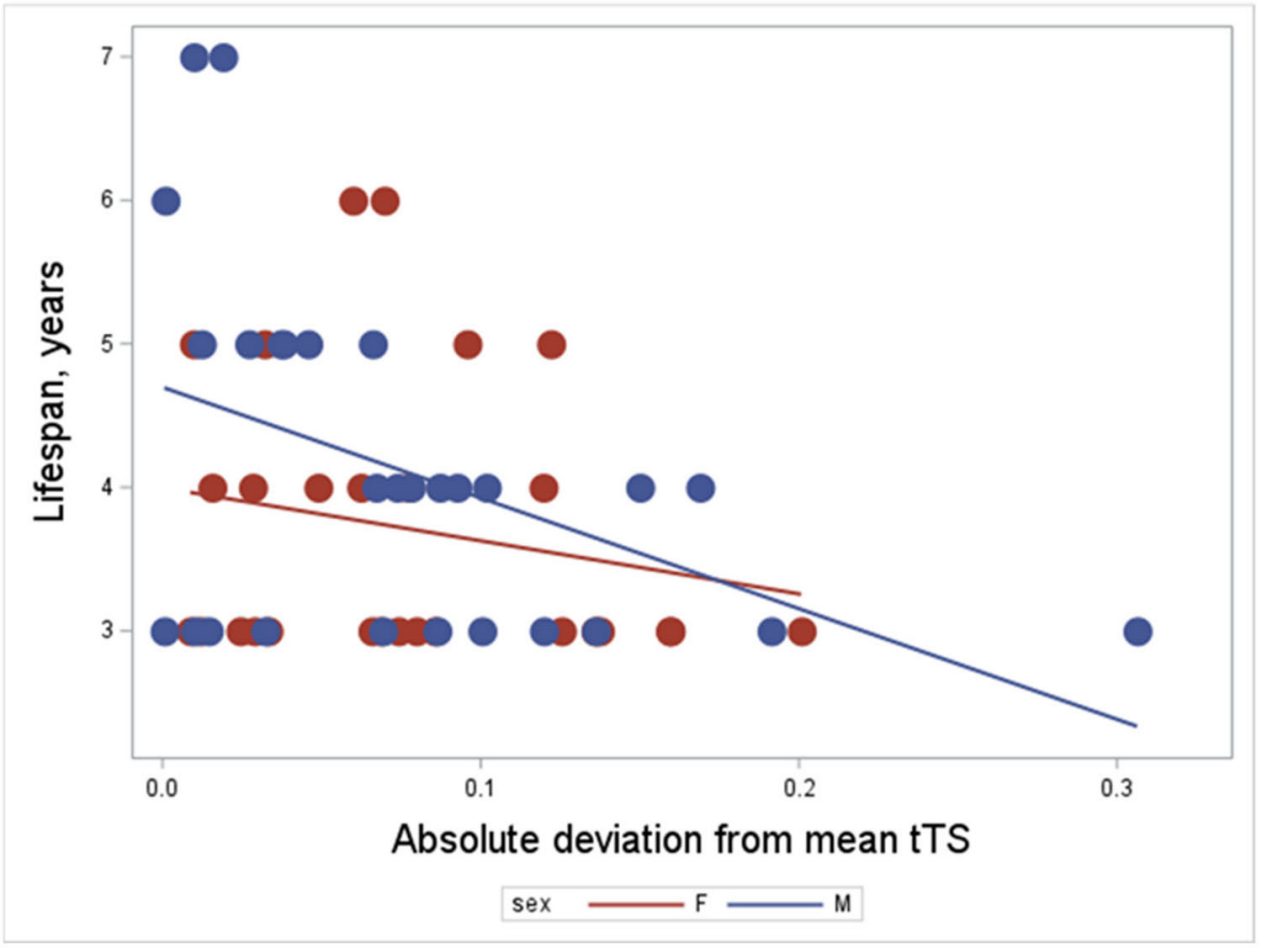
Testing for effects on lifespan of deviation from mean TL at hatching. Hatchling sex and mass were non‐significant in a first model and backwards‐eliminated from the final analysis. Hatchling telomere length per se was non‐significant (*p* = 0.354), while deviation from hatchling telomere mean was significant with a parameter value of −1.581 ± 0.532, SE (*t* = −2.97, *p* = 0.0029). [Color figure can be viewed at wileyonlinelibrary.com]

**Life span**: Our first analysis of a relationship between hatchling TLs, and their deviations from mean hatchling TL, included hatchling mass and sex. The latter two variables were non‐significant and a first logistic regression with negative binomial response distribution was under‐dispersed (Pearson's Chi‐square/DF = 0.27). We therefore reran the analysis (without the non‐significant hatchling mass and sex) as a generalized Poisson regression (Proc Countreg, SAS 9.4). The effect of hatchling TL per se on lifespan was non‐significant (*p* = 0.354), whereas deviation from mean TL significantly predicted lifespan with a parameter value of −1.581 ± 0.532, SE (*t* = −2.97, *p* = 0.0029; *N* = 56; Figure 2; Table 2). Because the two telomere traits show collinearity (which increases the risk of Type 1 error), we also tested their significance separately. Those tests confirmed the significant effect of deviation from mean TL on lifespan (*t* = −2.85, *p* = 0.0044), whereas TL itself remained non‐significant (*t* = 0.49, *p* = 0.622).

**Table 2:** Analysis of hatchling telomere effects on lifespan using Proc Countreg in SAS 9.4. The procedure applies a Conway Maxwell Poisson model and tests for overdispersion using the ‐ln(v) parameter (reported by default as _lnNU), which is significant (estimate = −1.52, standard error = 0.19, *t* = −7.89, *p* < 0.0001) confirming overdispersion and appropriate application of the Conway Maxwell procedure. tTShatch = log TS ratio at hatching, tTSHatchmeandev = log deviation from the population mean TSratio at hatching. DNA quality was non‐significant and backwards‐eliminated (*p* = 0.10).

Parameter estimates					
Parameter	DF	Estimate	Standard error	*t* Value	Approx Pr > |*t*|
Intercept	1	1.62	0.068	23.60	< 0.0001
tTSHatch	1	0.31	0.333	0.93	0.3541
tTSHatchmeandev	1	−1.58	0.532	−2.97	0.0029
_lnNu	1	−1.52	0.192	−7.89	< 0.0001

**Figure 3:**
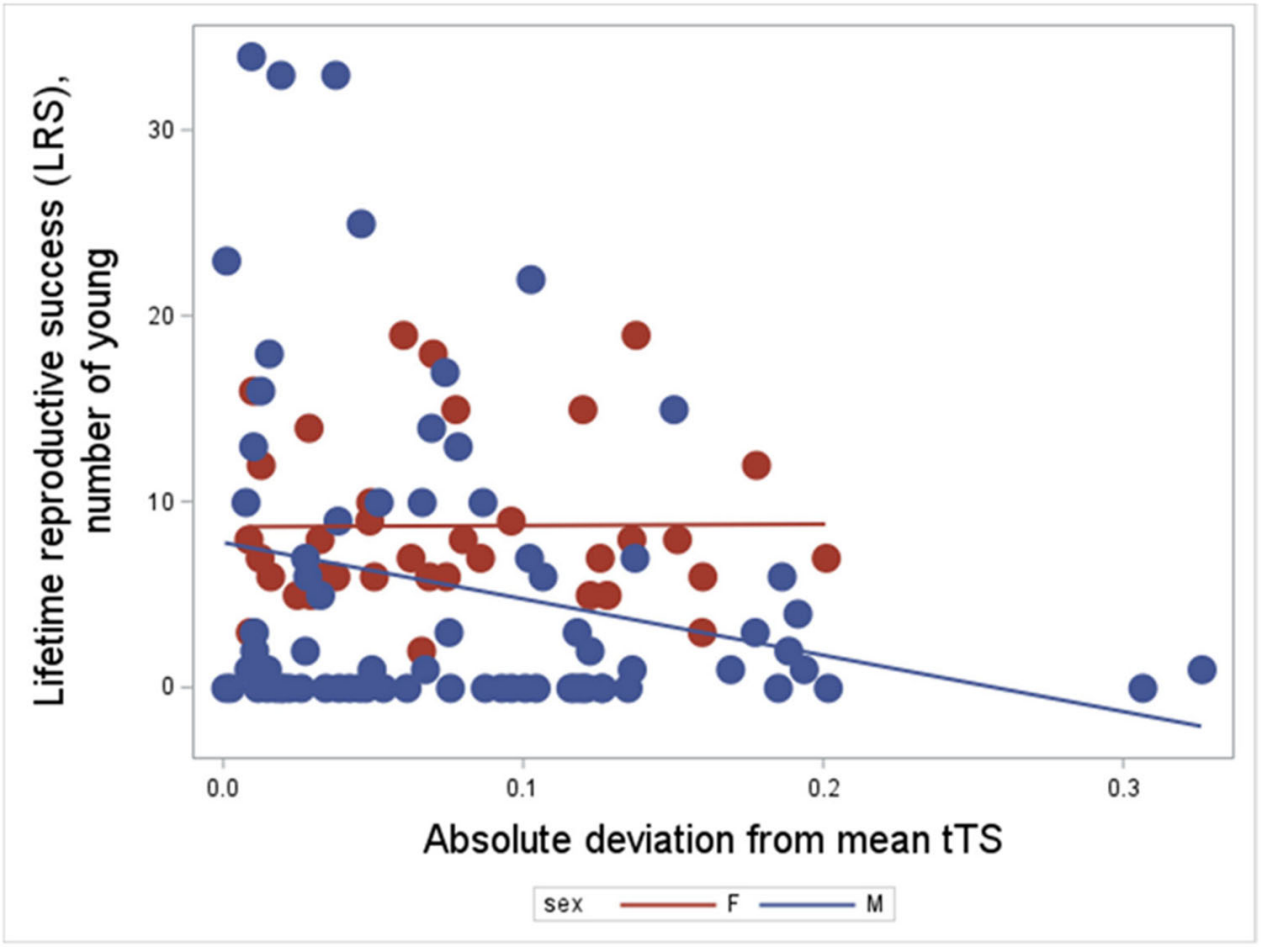
Effects of deviation from mean hatchling telomere length on lifetime reproductive success. Telomere length per se was non‐significant (*F*_1, 109_ = 0.08, *p* = 0.775), while the corresponding analysis of deviation from telomere means was significant (*F*_1, 109_ = 6.53, *p* = 0.012; parameter estimate = −19.3 ± 3.53; Table 3). [Color figure can be viewed at wileyonlinelibrary.com]

**Lifetime reproductive success:** We then analysed the complex effects of TL per se, and its deviation from the mean, on molecularly assigned LRS (cumulative counts of molecularly assigned offspring sired through life in males, cumulative fecundity in females). A first negative binomial regression using both variables and their interactions with sex, and birth year as random effect, showed significant relationships between both TL at hatching and its deviation from the population mean on LRS (Table 3). However, when their statistical significance was analysed separately, TL per se was a non‐significant predictor of LRS (*F* = 0.08, *p* = 0.775; Table 3), whereas deviation from the telomere mean length significantly predicted LRS (*F* = 6.53, *p* = 0.012; parameter estimate = −5.26 ± 2.06, SE; Figure 3; Table 3). Thus, the significant Type III effect of TL (*p* = 0.048) when analysed in co‐occurrence with the deviation from the mean may be a spurious effect and is not upheld in an independent significance test. In addition, the interaction between juvenile sex and absolute telomere deviation from the mean is significant with a positive regression coefficient, showing that females have ongoing selection for more variation than males (or that hatchling males show stronger effects of being closer to the mean for future LRS than do females).

**Table 3:** Modeling the relationship between log hatchling telomere length, and its deviation from the mean, and lifetime reproductive success. The sample size is 108 when sex is included in the analysis, with 35 female and 73 male hatchlings (3 additional hatchlings could not be sexed at hatching). A negative binomial regression was applied in Proc GLIMMIX, SAS 9.4. tTShatch = log hatchling telomere length, tTSHatchmeandev = log deviation from population mean telomere length at hatching. Birth year was used as random factor, which was significant with a log likelihood ratio test (*χ* ^2^ = 235, *p* < 0.001). The separate significance test of tTSHatch did not converge with birth year present in the model and is therefore presented on its own. DNA quality was nonsignificant and backwards‐eliminated (*p* = 0.11).

Type III tests of fixed effects					
Effect	Parameter estimate	Num DF	Den DF	*F* Value	Pr > *F* (Type III)
Intercept	3.50	0.687	9.97	5.09	0.0005
sex F	−1.68	0.722	93.6	−2.33	0.0221
sex M	0….				
tTSHatch	12.65	2.65	105	4.78	0.0052
tTSHatchmeandev	−19.32	3.54	105	−5.45	0.0154
tTSHatch*sex F	−11.02	5.04	97.26	−2.19	0.0312
tTSHatch*sex M	0….				
tTSHatchmeandev*sex F	20.95	6.32	90.33	3.32	0.0013
tTSHatchmeandev*sex M	0….				
*Note:* Separate significance testing of telomere length at hatching, and its deviation from the population mean (sex was excluded since *p* for sex was larger than 0.587 in both these analyses) with birth year as random effect.					

Type III tests of fixed effects				
Effect	Num DF	Den DF	*F* Value	Pr > *F*
tTSHatch	1	109	0.08	0.7747
tTSHatchmeandev	1	109	6.53	0.0120

**Figure 4:**
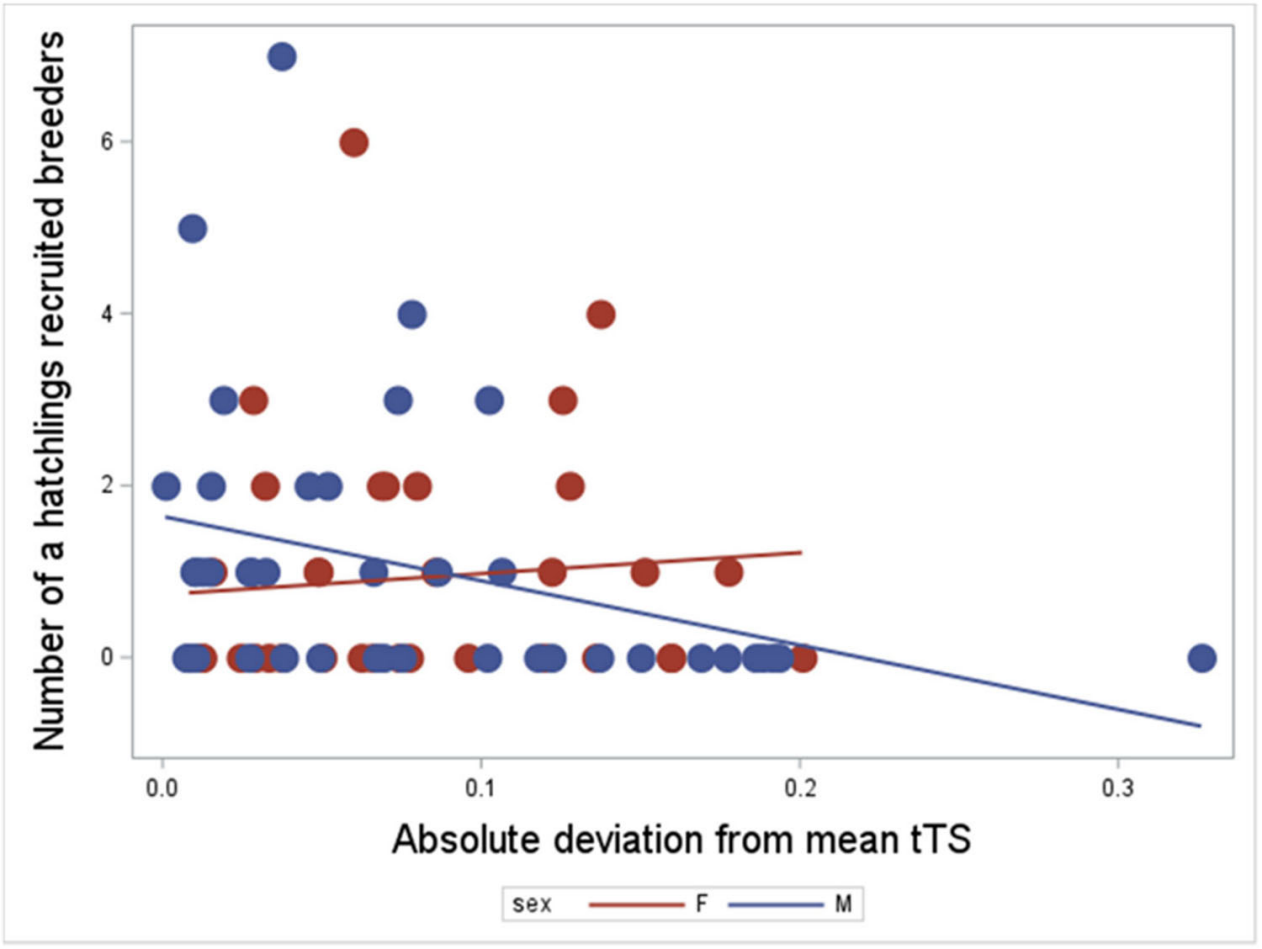
Effects of deviation from mean hatchling telomere length on the chance of recruitment of offspring to the reproducing population. The effects of telomere length at hatching on recruitment was non‐significant (*F*_1, 74_ = −3.25, *p* = 0.1220), while deviation from mean hatchling telomere length showed borderline significance (*F*_1, 74_ = −6.55, *p* = 0.0458). Separate reruns to minimize risk of collinearity showed similar results for telomere length per se (*p* = 0.0893), and deviation from the telomere mean (*p* = 0.0470; Table 4). [Color figure can be viewed at wileyonlinelibrary.com]

**Recruitment of reproducing young (recruits)**: Future recruitment rate of a hatchling's own offspring was not significantly related to its TL per se (*t* = −1.56, *p* = 0.1220; Table 4), but there was a borderline higher recruitment of future offspring from hatchlings that themselves had TLs closer to the mean (*t* = −2.03, *p* = 0.0458; birth year as random factor, Table 4). Separate significance testing to minimize risk of collinearity showed similar results for TL per se (*p* = 0.098), and deviation from the telomere mean (*p* = 0.0470; Figure 4; Table 4).

**Table 4:** Negative binomial regression in Proc GLIMMIX of the relationships between log telomere length at hatching and its deviation from the mean on number of confirmed recruited offspring into the breeding population as adults. Sex was not significant and was excluded from the analysis. Birth year was used as a random factor and DNA quality was non‐significant and backwards‐eliminated (*p* = 0.15).

Parameter estimates					
**Effect**	**Estimate**	**Standard error**	**DF**	***t* Value**	**Pr** >** |*t*** |
Intercept	0.026	0.376	71.9	0.07	0.9442
tTSHatch	−3.25	2.081	74	−1.56	0.1220
tTSHatchmeandev	−6.55	3.226	74	−2.03	0.0458
*Note:* Separate significance testing of telomere length at hatching, and its deviation from the population mean					

Type III tests of fixed effects				
Effect	Num DF	Den DF	*F* Value	Pr > *F*
tTSHatch	1	75	2.96	0.0893
tTSHatchmeandev	1	75	3.01	0.0470

### 3.3 The Cost of Telomere Elongation for Hatchlings With Short Telomeres

An analysis of the difference in TL change from hatching to the first observation as adults showed that lizards with elongating telomeres had about twice as much variation in TL change (D) as did those that showed telomere shortening (equality of variance: *F*_63, 49_ = 23.11, *p* < 0.0001; Table 5). This disparity encouraged further analyses of factors associated with elongation. Overall, the correlation coefficient between a lizard's first (hatchling) and later (adult) TL was *r* = 0.199 (*p* = 0.036, *N* = 112). The correlation between TL as hatchling and adult while controlling for age in a partial correlation was *r* = 0.196, *N* = 112, *p* = 0.039). The correlation between TL as adult and age was *r* = 0.049, *N* = 112, *p* = 0.604. Thus, there is a slight effect of TL at hatching on TL as adult but there is no effect of age per se on TL. This supports our previous analyses which shows no age effect on TLs in sand lizards (e.g., Olsson et al. 2011, 2024).

**Table 5:** Using Proc TTest, SAS 9.4 to test for equality of variances between lizards that elongated their telomeres (“pos”) versus those that did not change or showed net telomere attrition (“neg”). These analyses are based on the following numbers of sexes and age groups: Females: 2 years, *N* = 31, 3 years *N* = 6. Males: 2 years *N* = 69, 3 years *N* = 4, 4 years *N* = 2. Total sample size = 112.

Change in telomere length	*N*	Mean	Std Dev	Std Err	Min	Max
Neg	63	0.13	0.075	0.0095	0.0014	0.336
Pos	49	−0.16	0.133	0.019	−0.474	−0.009

Equality of variances				
Method	Num DF	Den DF	*F* Value	Pr > *F*
Folded F	48	62	3.11	< 0.0001

However, the average TL at maturity was the same for lizards that hatched with “short telomeres” (i.e., shorter than the population mean) as for those that hatched with “long telomeres,” controlling for individual ID as a random effect (i.e., taking into account that we had multiple observations as adults for some individuals; Table 6). We include age in the Table 6 analysis for completion; when it is backwards‐eliminated the *p* value of hatchling TL category effect on adult TL increases further to 0.634.

**Table 6:** GLMM results from a Proc MIXED in SAS 9.4 analysing to what extent individuals that hatch out with telomeres longer (“Hatchlongies”) or shorter (“Hatchshorties”) than the population mean differ in their telomere length as adults (there was no such effect, *p* = 0.561). Age consistently show no relation to telomere length in our analyses. We control for pseudoreplication when having more than one observation as adults by including individual number as a random factor. DNA quality was non‐significant (*p* = 0.619) and backwards‐eliminated.

Response: Log telomere length as adult					
Effect tTSHatchClass	Estimate	Standard error	DF	*t* Value	Pr > |*t*|
Intercept	−0.045	0.037	179	−1.22	0.223
tTSHatchClass Hatchlongies	0.016	0.027	118	0.58	0.561
tTSHatchClass Hatchshorties	0….				
Age	−0.017	0.013	176	−1.30	0.194

**Figure 5:**
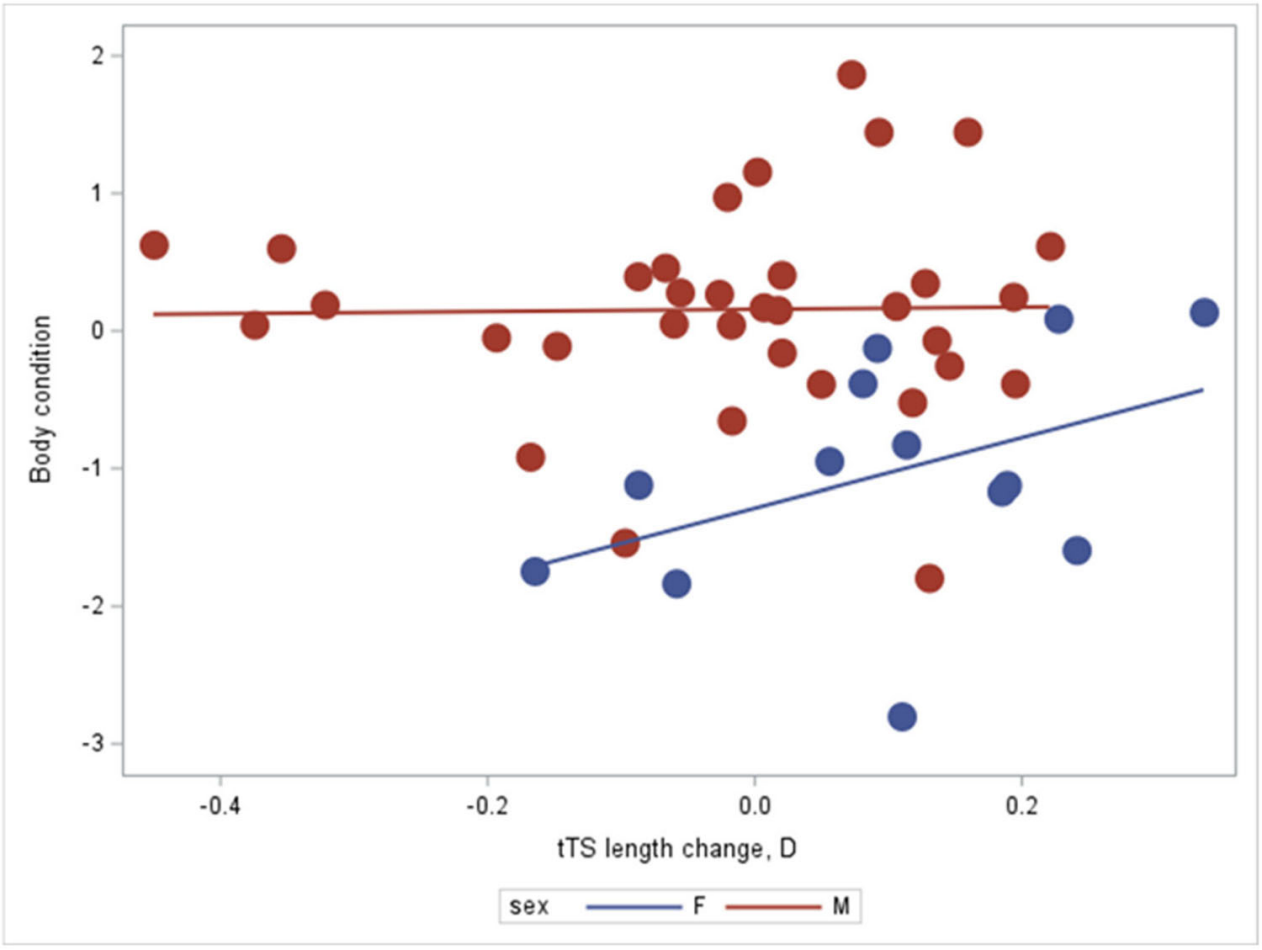
Hatchlings with telomeres longer than the mean showed no effect on body condition from telomere length change from their first (at hatching) to the first telomere recording as adults (parameter estimate = 0.58 ± 0.72, mean ± SE, *N* = 47, *t* = 0.81, *p* = 0.423), while controlling for sex (*p* < 0.0001). [Color figure can be viewed at wileyonlinelibrary.com]

We then focused on differences in telomere dynamics between hatchlings with telomeres longer *versus* shorter than the mean, to explore the prediction that mechanisms to repair and elongate TL incur metabolic costs. Mechanisms to actively shorten telomeres seem less likely, given that TL inevitably will reduce during cell divisions. Our next set of analyses, therefore, investigated whether TL elongation through life involves costly investments into TL repair and elongation great enough to affect overall body condition. We would expect such an effect particularly in “short telomere” hatchlings (measured as the disparity between hatching and first adult TL estimates while controlling for regression to the mean [the “D” parameter; Verhulst et al. 2013]). By the time they attained maturity, individuals with “long telomeres” at hatching showed no significant effect on adult body condition of the magnitude of telomere change during juvenile life (Generalized Linear Model, Proc GLM in SAS 9.4; parameter estimate = 0.58 ± 0.72, SE, DF = 1, *t* = 0.81, *N* = 47, *p* = 0.423; Figure 5).

**Figure 6:**
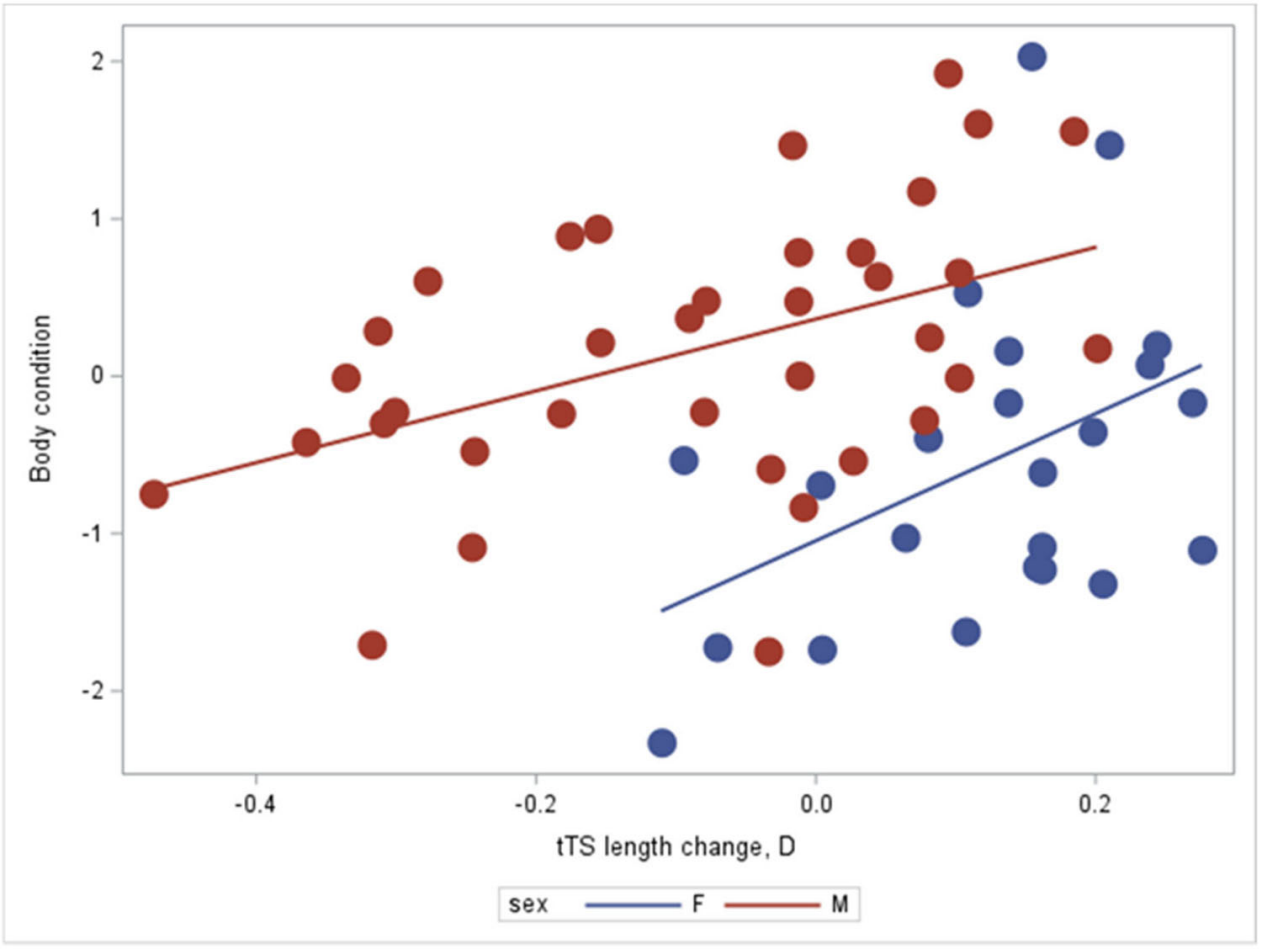
For hatchlings with telomeres shorter than the mean, telomere elongation (i.e., negative D), was a significant predictor of body condition in both males and females (parameter estimate = 2.52 ± 1.02, SE, *N* = 59 *t* = 2.64, *p* = 0.0006). While the slopes were the same for both sexes, body condition was lower in females (*p* = < 0.0001; details in Table 7). [Color figure can be viewed at wileyonlinelibrary.com]

For hatchlings with “short telomeres,” however, a greater degree of telomere elongation during juvenile life was associated with lower body condition in both males and females (parameter estimate = 2.64 ± 0.73, DF = 1, *t* = 3.62, *N* = 59, *p* = 0.00006; Figure 6). Although the slopes were similar for both sexes, mean body condition was lower in females both at hatching (estimate: −1.27 ± 0.27, SE, *p* < 0.0001; details in Table 7) and over the entire investment spectrum into telomere repair (*p* = 0.0006; Figure 6; Table 7; in Table 7 we include age for completeness even though it would be more appropriate to exclude this from the analysis, since it has no effect). In a complementary analysis, we assessed whether hatchlings with “long” *versus* “short” telomeres differed in body condition at hatching, which would suggest different resource levels for somatic maintenance before hatching. No such difference was found (Proc Mixed, Num DF = 1, Den DF = 81, *F* = 0.99, *p* = 0.324).

**Table 7:** Generalized Linear Model in SAS Proc GLM of the relationship between telomere dynamics and body condition at first observation as adult in lizards hatching out with telomeres shorter than the population mean. Body condition is estimated as residuals from a snout‐vent length—body mass regression. D = telomere change controlling for regression to the mean (following Sudyka et al. 2019). Age is included for completeness and to remove any ambiguity of its importance (the more appropriate results are ignoring age in the analysis and are provided in the body of the text). *N* = 59. SAS sets one category to zero by default against which other categories are contrasted. DNA quality was non‐significant (*p* = 0.566) and backwards‐eliminated. Age was also run as a continuous covariate in a separate model and was also then found non‐significant (*p* = 0.276).

Response variable: Body condition at first observation as adult				
Parameter	Estimate	Standard error	*t* Value	Pr > |*t*|
Intercept	0.71	0.848	0.83	0.408
Sex F	−1.29	0.277	−4.64	< 0.0001
Sex M	0.0…			
D (the lower D is, the more have the telomeres elongated)	2.61	0.738	3.53	0.0009
Age 2	−0.35	0.865	−0.41	0.686
Age 3	0.10	0.943	0.11	0.916
Age 4	0.0…			

## 4 Discussion

We show that natural selection acts on hatchling TL in a population of a free‐ranging reptile. Hatchling sand lizards with telomeres close to the population mean length had longer lifespans, higher LRS and better offspring recruitment rate than did conspecifics with shorter or longer telomeres. Presumably reflecting that adaptive advantage, hatchlings that begin life with shorter‐than‐average telomeres compensated by increasing TL during juvenile life—even though that response incurred a cost to body condition as adults.

Elsewhere (Olsson et al. 2024; Olsson et al. 2011) we have shown that telomere repair and maintenance in adult sand lizards is costly, sex‐specific and strongly impacted by resource abundance (Olsson et al. 2024); we exploited a “pseudo‐experimental” situation that emerges after attacks by predators when lizards autotomise (“drop”) their tails and thereby lose resources stored in the tail (Olsson et al. 2024). Females with higher reproductive output, and those that had lost their tails, showed less telomere elongation. In both sexes, adult lizards with longer telomeres live for longer, but the link to LRS differed between the sexes; males with more telomere elongation had higher LRS whereas adult females with higher lifetime reproductive investment showed more telomere attrition (Olsson et al. 2024). In combination with the current study, hatchlings and adult sand lizards thus appear to differ in the relationships between telomeres and the fitness components lifespan, LRS and recruitment of future breeders.

This ontogenetic difference calls for a comparison of life history investment patterns in hatchlings and adults, and how allocation to telomere maintenance *versus* other resource‐demanding activities, such as growth, may differ between the two life‐history stages. It is well known that growth patterns in Squamate reptiles (lizards and snakes) is characterized by intense growth in early life, followed by tapering‐off of growth rate, which may persist to some extent throughout life (Avery 1994). Furthermore, during the early part of life, stabilizing selection is commonly observed on other phenotypic traits such as body mass (Adriaensen et al. 1998; Sanjak et al. 2018), with fitness repercussions later in life. Such ontogenetic variations in investment into growth, somatic maintenance and future reproduction are consistent with classic life history theory (Stearns 1976) as well as the metabolic theory of ecology (Mauritsson and Jonsson 2023) and dynamic energy budgeting theory (Kooijman 2014; Kooijman and Lika 2014). These approaches model differential investments into metabolic organization through life, such as age‐specific fat‐body investments in honeybee workers (*Apis mellifera*) (Seehuus et al. 2013). Thus, young sand lizards may be under selection to maximize somatic growth and optimize investment into telomere maintenance, whereas adults that are much closer to arrested growth allocate more resources into sex‐specific telomere repair and maintenance traded off against reproductive effort. Similar scenarios may explain effects of telomere patterns early in ontogeny on later reproductive success observed in other taxa, such as fairy wrens (*Melospiza* sp.) (Eastwood et al. 2022; Eastwood et al. 2023) and zebra finches (*Taeniopygia guttata*) (Heidinger et al. 2012). From this perspective, our results support the Casagrande and Hau (Casagrande and Hau 2019) “metabolic attrition/elongation” hypothesis of telomere dynamics, whereby TL is maintained at a cost in species with TL repair mechanisms. Below, we address a series of questions related to our findings.

Our study did not address the underlying genetic or physiological mechanisms that underly the ability of juvenile sand lizards to facultatively increase their TLs, but published literature provides suggestions in this respect. Several pathways may be involved; for example, glucocorticoids (GC) that increase during stress (Casagrande and Hau 2019; Casagrande et al. 2023; Casagrande et al. 2020) increase telomerase gene expression, such that stressed laboratory rats had 54% higher telomerase levels than did non‐stressed rats (Beery et al. 2012). Likewise, the mechanisms that result in loss of body condition in lizards that increase their TLs ontogenetically are unclear. The metabolic attrition hypothesis (Casagrande and Hau 2019) predicts increased attrition during anabolic processes, influenced by rapid growth and other resource‐demanding maintenance processes impacted by GCs and sex steroids (Casagrande and Hau 2019; Casagrande et al. 2023; Casagrande et al. 2020).

Perhaps the most fundamental unanswered question arising from our study involves the causation of stabilizing selection on TL. Why do lizards with unusually short or unusually long telomeres at hatching have lower fitness than do conspecifics with telomeres close to the population‐average length? Multiple reports have linked shorter telomeres to ageing (e.g., Chan and Blackburn 2004; Epel et al. 2004; McEachern et al. 2000; Monaghan and Haussmann 2006), offering a possible explanation for the fitness disadvantage of shorter‐than‐average telomeres, and hence for intense selection for facultative adjustment of TL in young lizards facing this problem. Why there is a disadvantage to longer‐than‐average telomeres is more difficult to explain, leading some researchers to suggest that TL and rate of attrition are best viewed as indirect biomarkers of overall vigor and viability, rather as targets of selection per se (Kappei and Londoño‐Vallejo 2008). One possibility is that high levels of expression of telomerase—the main mechanism that increases TL—may have deleterious consequences. For example, telomerase can facilitate the growth of cancer tumors (a Scopus search of “telomerase AND disease” on May 20, 2025 produced 91,803 hits). If such telomerase‐enhanced disease impacts LRS, that fitness penalty could impose strong selection against the mechanism that produces telomere elongation (Madsen et al. 2017; Ujvari et al. 2022).

More generally, we make two fundamental observations in this study. First, costly investment strategies appear to be in place to maintain and elongate telomeres in young lizards that hatch with shorter‐than‐average TLs, despite the costs of a reduction in body condition later in life. Second, selection appears to favor an “optimal” rather than a maximal TL through early life. Our data suggest that TLs within a population of free‐ranging lizards are under stabilizing selection, creating a selective advantage to individuals that are capable of facultatively adjusting TL towards the population mean value. Decreasing TL may not require specific control mechanisms, because of natural attrition during successive cycles of cell division; but TL elongation requires the production of an enzyme (telomerase) that is likely to have negative impacts on viability if expressed at high levels. Such ancillary costs might also contribute to the observed reduction in body condition of lizards that invest in telomere elongation during juvenile life. Presumably the fitness advantage of optimal TL outweighs the costs of telomere elongation in our lizard population, but it is easy to imagine a situation whereby costs of telomere elongation outweigh benefits. In such cases, we would expect the facultative ontogenetic adjustment of TL to be replaced by a less flexible system in which maximal TL is set early in life and decreases thereafter except in circumstances where TL reaches critically low thresholds.

## 5 Conclusions

The complex pattern of telomerase expression seen in endothermic vertebrates may reflect a general loss of the ability to elongate telomeres because costs of high telomerase levels usually exceed benefits; but with the retention of that ability in cases where the fitness penalties of telomere truncation are especially severe. Future field‐based research could usefully explore selection on TL in endothermic species that retain the ancestral ability to manipulate TLs during ontogeny.

## Author Contributions

M.O. designed the study, conducted field work, statistical analyses and wrote the first draft of the manuscript. E.W., E.M., N.R., and R.S. contributed to field and laboratory data, analyses and writing. All authors read and commented on a first draft of the manuscript by Mats Olsson.

## Ethics Statement

The research described in this manuscript was performed under a sequence of permits from the ethics committee at University of Gothenburg, Sweden, of which the latest was Dnr 5.8.18‐04505/2025. All field and laboratory work on sand lizards (*Lacerta agilis*) have been approved in a sequence of permits issued by the Nature Conservation Council (“Länsstyrelsen” in Swedish) in the province of Halland, the latest being 1300–2025.

## Consent

The authors have nothing to report.

## Conflicts of Interest

The authors declare no conflicts of interest.

## Data Availability

The data are available at Dryad http://datadryad.org/share/qI1GAHKlkZp5Q8NM0FRGdxKO7DUGcIAh1LlsqHrZY6c.

## References

[ede70020-bib-0001] Adriaensen, F. , A. Dhondt , S. V. Dongen , L. Lens , and E. Matthysen . 1998. “Stabilizing Selection on Blue Tit Fledgling Mass in the Presence of Sparrowhawks.” Proceedings of the Royal Society of London. Series B: Biological Sciences 265, no. 1400: 1011–1016. 10.1098/rspb.1998.0392.

[ede70020-bib-0002] Agata, A. , and T. Nomura . 2024. “Thermal Adaptations in Animals: Genes, Development, and Evolution.” Advances in Experimental Medicine and Biology 1461: 253–265. 10.1007/978-981-97-4584-5_18. 39289287

[ede70020-bib-0003] Anderholm, S. , M. Olsson , E. Wapstra , and K. Ryberg . 2004. “Fit and Fat From Enlarged Badges: A Field Experiment on Male Sand Lizards.” Proceedings of the Royal Society of London Series B‐Biological Sciences 271: S142–S144. 10.1098/rsbl.2003.0094 PMC1810037 15252966

[ede70020-bib-0004] Aubert, G. , G. M. Baerlocher , I. Vulto , S. S. Poon , and P. M. Lansdorp . 2012. “Collapse of Telomere Homeostasis in Hematopoietic Cells Caused by Heterozygous Mutations in Telomerase Genes.” PLoS Genetics 8, no. 5: e1002696. 10.1371/journal.pgen.1002696. 22661914 PMC3355073

[ede70020-bib-0005] Avery, R. A. 1994. “Growth in Reptiles.” Gerontology 40, no. 2–4: 193–199. 10.1159/000213587. 7926856

[ede70020-bib-0006] Axelsson, J. , E. Wapstra , E. Miller , N. Rollings , and M. Olsson . 2020a. “Contrasting Seasonal Patterns of Telomere Dynamics in Response to Environmental Conditions in the Ectothermic Sand Lizard, Lacerta Agilis.” Scientific Reports 10, no. 1: 182. 10.1038/s41598-019-57084-5. 31932620 PMC6957525

[ede70020-bib-0007] Axelsson, J. , E. Wapstra , E. Miller , N. Rollings , and M. Olsson . 2020b. “Contrasting Seasonal Patterns of Telomere Dynamics in Response to Environmental Conditions in the Ectothermic Sand Lizard, Lacerta Agilis.” Scientific Reports 10, no. 1: 1–9. 31932620 10.1038/s41598-019-57084-5 PMC6957525

[ede70020-bib-0008] Baird, D. 2007. “Telomeres II.” Experimental Gerontology 43, no. 1: 15–19. 10.1016/j.exger.2007.10.002. 17981417

[ede70020-bib-0009] Barrett, E. L. B. , and D. S. Richardson . 2011. “Sex Differences in Telomeres and Lifespan.” Aging cell 10, no. 6: 913–921. 10.1111/j.1474-9726.2011.00741.x. 21902801

[ede70020-bib-0010] Beery, A. K. , J. Lin , J. S. Biddle , D. D. Francis , E. H. Blackburn , and E. S. Epel . 2012. “Chronic Stress Elevates Telomerase Activity in Rats.” Biology Letters 8, no. 6: 1063–1066. 10.1098/rsbl.2012.0747. 23054915 PMC3497144

[ede70020-bib-0011] Bénard, C. , and S. Hekimi . 2002. “Long‐Lived Mutants, the Rate of Aging, Telomeres and the Germline in *Caenorhabditis Elegans* .” Mechanisms of Ageing and Development 123, no. 8: 869–880. 12044935 10.1016/s0047-6374(02)00024-6

[ede70020-bib-0012] Blasco, A. 2003. “Telomeres in Cancer and Aging: Lessons From the Mouse.” Cancer Letters 194, no. 2: 183–188. 10.1016/S0304-3835(02)00705-X. 12757976

[ede70020-bib-0013] Burraco, P. , P. M. Lucas , and P. Salmón . 2022. “Telomeres in a Spatial Context: A Tool for Understanding Ageing Pattern Variation in Wild Populations.” Ecography 2022, no. 6: e05565. 10.1111/ecog.05565.

[ede70020-bib-0014] Casagrande, S. , and M. Hau . 2019. “Telomere Attrition: Metabolic Regulation and Signalling Function?” Biology Letters 15, no. 3: 20180885. 10.1098/rsbl.2018.0885. 30890069 PMC6451386

[ede70020-bib-0015] Casagrande, S. , J. L. Loveland , M. Oefele , et al. 2023. “Dietary Nucleotides Can Prevent Glucocorticoid‐Induced Telomere Attrition in a Fast‐Growing Wild Vertebrate.” Molecular Ecology 32, no. 19: 5429–5447. 10.1111/mec.17114. 37658759

[ede70020-bib-0016] Casagrande, S. , A. Stier , P. Monaghan , et al. 2020. “Increased Glucocorticoid Concentrations in Early Life Cause Mitochondrial Inefficiency and Short Telomeres.” Journal of Experimental Biology 223, no. 15: jeb222513. 10.1242/jeb.222513. 32532864

[ede70020-bib-0017] Cawthon, R. M. 2002. “Telomere Measurement by Quantitative PCR.” Nucleic Acids Research 30, no. 10: 47e. 12000852 10.1093/nar/30.10.e47 PMC115301

[ede70020-bib-0018] Chan, S. R. W. L. , and E. H. Blackburn . 2004. “Telomeres and Telomerase.” Philosophical Transactions of the Royal Society of London. Series B: Biological Sciences 359: 109–122. 15065663 10.1098/rstb.2003.1370 PMC1693310

[ede70020-bib-0019] Chik, H. Y. J. , A. M. Sparks , J. Schroeder , and H. L. Dugdale . 2022. “A Meta‐Analysis on the Heritability of Vertebrate Telomere Length.” Journal of Evolutionary Biology 35, no. 10: 1283–1295. 10.1111/jeb.14071. 35932478 PMC9804776

[ede70020-bib-0020] Criscuolo, F. , P. Bize , L. Nasir , et al. 2009. “Real‐Time Quantitative PCR Assay for Measurement of Avian Telomeres.” Journal of Avian Biology 40, no. 3: 342–347.

[ede70020-bib-0021] Eastwood, J. R. , T. Connallon , K. Delhey , et al. 2022. “Hot and Dry Conditions Predict Shorter Nestling Telomeres in an Endangered Songbird: Implications for Population Persistence.” Proceedings of the National Academy of Sciences 119, no. 25: e2122944119. 10.1073/pnas.2122944119. PMC9231487 35696588

[ede70020-bib-0022] Eastwood, J. R. , A. Dupoué , K. Delhey , S. Verhulst , A. Cockburn , and A. Peters . 2023. “When Does Early‐Life Telomere Length Predict Survival? A Case Study and Meta‐Analysis.” Molecular Ecology 32, no. 11: 3000–3013. 10.1111/mec.16894. 36811398

[ede70020-bib-0023] Epel, E. S. , E. H. Blackburn , J. Lin , et al. 2004. “Accelerated Telomere Shortening in Response to Life Stress.” Proceedings of the National Academy of Sciences 101, no. 49: 17312–17315. 10.1073/pnas.0407162101 PMC534658 15574496

[ede70020-bib-0024] Fitzpatrick, L. J. , M. Olsson , A. Pauliny , G. M. While , and E. Wapstra . 2021. “Individual Telomere Dynamics and Their Links to Life History in a Viviparous Lizard.” Proceedings of the Royal Society B: Biological Sciences 288, no. 1951: 20210271. 10.1098/rspb.2021.0271. PMC8150041 34034513

[ede70020-bib-0025] Gillooly, J. F. , J. P. Gomez , and E. V. Mavrodiev . 2017. “A Broad‐Scale Comparison of Aerobic Activity Levels in Vertebrates: Endotherms Versus Ectotherms.” Proceedings of the Royal Society B: Biological Sciences 284, no. 1849: 20162328. 10.1098/rspb.2016.2328. PMC5326522 28202808

[ede70020-bib-0026] Glenn, A. , and C. E. Armstrong . 2019. “Physiology of Red and White Blood Cells.” Anaesthesia & Intensive Care Medicine 20, no. 3: 170–174. 10.1016/j.mpaic.2019.01.001.

[ede70020-bib-0027] Glousker, G. , A.‐S. Briod , M. Quadroni , and J. Lingner . 2020. “Human Shelterin Protein POT1 Prevents Severe Telomere Instability Induced by Homology‐Directed DNA Repair.” The EMBO Journal 39, no. 23: e104500. 10.15252/embj.2020104500. 33073402 PMC7705456

[ede70020-bib-0028] Heidinger, B. J. , J. D. Blount , W. Boner , K. Griffiths , N. B. Metcalfe , and P. Monaghan . 2012. “Telomere Length in Early Life Predicts Lifespan.” Proceedings of the National Academy of Sciences 109, no. 5: 1743–1748. 10.1073/pnas.1113306109. PMC3277142 22232671

[ede70020-bib-0029] Heidinger, B. J. , K. A. Herborn , H. M. V. Granroth‐Wilding , et al. 2016. “Parental Age Influences Offspring Telomere Loss.” Functional Ecology 30, no. 9: 1531–1538. 10.1111/1365-2435.12630.

[ede70020-bib-0031] Hertzog, R. G. 2006. “Ancestral Telomere Shortening: A Countdown That Will Increase Mean Life Span?” Medical Hypotheses 67, no. 1: 157–160. 10.1016/j.mehy.2006.01.034. 16530337

[ede70020-bib-0032] Hoelzl, F. , J. S. Cornils , S. Smith , Y. Moodley , and T. Ruf . 2016. “Telomere Dynamics in Free‐Living Edible Dormice (Glis Glis): The Impact of Hibernation and Food Supply.” Journal of Experimental Biology 219, no. Pt 16: 2469–2474. 10.1242/jeb.140871. 27535986 PMC5004978

[ede70020-bib-0033] Jemielity, S. , M. Kimura , K. M. Parker , et al. 2007. “Short Telomeres in Short‐Lived Males: What Are the Molecular and Evolutionary Causes?” Aging Cell 6, no. 2: 225–233. 10.1111/j.1474-9726.2007.00279.x. 17346255 PMC1859884

[ede70020-bib-0034] Jia, P. , C. Her , and W. Chai . 2015. “DNA Excision Repair at Telomeres.” DNA Repair 36: 137–145. 10.1016/j.dnarep.2015.09.017. 26422132 PMC4688237

[ede70020-bib-0035] Kappei, D. , and J. A. Londoño‐Vallejo . 2008. “Telomere Length Inheritance and Aging.” Mechanisms of Ageing and Development 129, no. 1–2: 17–26. 10.1016/j.mad.2007.10.009. 18054991

[ede70020-bib-0036] Kärkkäinen, T. , M. Briga , T. Laaksonen , and A. Stier . 2022. “Within‐Individual Repeatability in Telomere Length: A Meta‐Analysis.” Molecular Ecology 31, no. 23: 6339–6359. 10.1111/mec.16155. 34455645

[ede70020-bib-0037] Kooijman, S. A. L. M. 2014. “Metabolic Acceleration in Animal Ontogeny: An Evolutionary Perspective.” Journal of Sea Research 94: 128–137. 10.1016/j.seares.2014.06.005.

[ede70020-bib-0038] Kooijman, S. A. L. M. , and K. Lika . 2014. “Resource Allocation to Reproduction in Animals.” Biological Reviews 89, no. 4: 849–859. 10.1111/brv.12082. 24517882

[ede70020-bib-0039] Lande, R. , and S. J. Arnold . 1983. “The Measurement of Selection on Correlated Characters.” Evolution 37, no. 6: 1210–1226. 10.2307/2408842. 28556011

[ede70020-bib-0040] Lanna, A. , B. Vaz , C. D'Ambra , et al. 2022. “An Intercellular Transfer of Telomeres Rescues T Cells From Senescence and Promotes Long‐Term Immunological Memory.” Nature Cell Biology 24, no. 10: 1461–1474. 10.1038/s41556-022-00991-z. 36109671 PMC7613731

[ede70020-bib-0041] Lansdorp, P. 2022a. “Telomere Length Regulation [Review].” Frontiers in Oncology 12: 943622. 10.3389/fonc.2022.943622. 35860550 PMC9289283

[ede70020-bib-0042] Lansdorp, P. M. 2022b. “Sex Differences in Telomere Length, Lifespan, and Embryonic Dyskerin Levels.” Aging Cell 21, no. 5: e13614. 10.1111/acel.13614. 35441417 PMC9124296

[ede70020-bib-0043] Laurell, H. , J. S. Iacovoni , and A. Abot , et al. 2012. “Correction of RT–qPCR Data for Genomic DNA‐Derived Signals With Validprime.” Nucleic Acids Research 40, no. 7: e51. 10.1093/nar/gkr1259. 22228834 PMC3326333

[ede70020-bib-0044] Madsen, T. , A. Arnal , M. Vittecoq , et al. 2017. “Cancer Prevalence and Etiology in Wild and Captive Animals.” In Ecology and Evolution of Cancer, 11–46. 10.1016/B978-0-12-804310-3.00002-8.

[ede70020-bib-0045] Mannherz, W. , and S. Agarwal . 2023. “Thymidine Nucleotide Metabolism Controls Human Telomere Length.” Nature Genetics 55, no. 4: 568–580. 10.1038/s41588-023-01339-5. 36959362 PMC11000509

[ede70020-bib-0046] Matsubara, K. , Y. Uno , K. Srikulnath , Y. Matsuda , E. Miller , and M. Olsson . 2015. “No Interstitial Telomeres on Autosomes but Remarkable Amplification of Telomeric Repeats on the W Sex Chromosome in the Sand Lizard (Lacerta Agilis).” Journal of Heredity 106, no. 6: 753–757. 10.1093/jhered/esv083. 26464091

[ede70020-bib-0047] Mauritsson, K. , and T. Jonsson . 2023. “A New Flexible Model for Maintenance and Feeding Expenses That Improves Description of Individual Growth in Insects.” Scientific Reports 13, no. 1: 16751. 10.1038/s41598-023-43743-1. 37798309 PMC10556006

[ede70020-bib-0048] McEachern, M. J. , A. Krauskopf , and E. H. Blackburn . 2000. “Telomeres and Their Control.” Annual Review of Genetics 34: 331–358. 10.1146/annurev.genet.34.1.331 11092831

[ede70020-bib-0049] McLennan, D. , J. D. Armstrong , D. C. Stewart , et al. 2018. “Telomere Elongation During Early Development Is Independent of Environmental Temperatures in Atlantic Salmon.” Journal of Experimental Biology 221, no. 11: jeb178616. 10.1242/jeb.178616. 29636409 PMC6031317

[ede70020-bib-0050] McLennan, D. , H. Recknagel , K. R. Elmer , and P. Monaghan . 2019. “Distinct Telomere Differences Within a Reproductively Bimodal Common Lizard Population.” Functional Ecology 33, no. 10: 1917–1927. 10.1111/1365-2435.13408. 31762528 PMC6853248

[ede70020-bib-0051] Monaghan, P. 2024. “Linking Telomere Dynamics to Evolution, Life History and Environmental Change: Perspectives, Predictions and Problems.” Biogerontology 25, no. 2: 301–311. 10.1007/s10522-023-10081-8. 38252370 PMC10998769

[ede70020-bib-0052] Monaghan, P. , and M. F. Haussmann . 2006. “Do Telomere Dynamics Link Lifestyle and Lifespan?” Trends in Ecology & Evolution 21, no. 1: 47–53. 16701469 10.1016/j.tree.2005.11.007

[ede70020-bib-0053] Nussey, D. H. , D. Baird , E. Barrett , et al. 2014. “Measuring Telomere Length and Telomere Dynamics in Evolutionary Biology and Ecology.” Methods in Ecology and Evolution 5, no. 4: 299–310. 10.1111/2041-210x.12161. 25834722 PMC4375921

[ede70020-bib-0054] Olsson, M. 1993. “Male Preference for Large Females and Assortative Mating for Body Size in the Sand Lizard (Lacerta Agilis).” Behavioral Ecology and Sociobiology 32: 337–341.

[ede70020-bib-0055] Olsson, M. 1994. “Nuptial Coloration in the Sand Lizard, *Lacerta Agilis*: An Intra‐Sexually Selected Cue to Fighting Ability.” Animal Behaviour 48, no. 3: 607–613. 10.1006/anbe.1994.1280.

[ede70020-bib-0056] Olsson, M. , B. Bererhi , E. Miller , et al. 2022. “Inbreeding Effects on Telomeres in Hatchling Sand Lizards (Lacerta Agilis): An Optimal Family Affair?” Molecular Ecology 31, no. 24: 6605–6616. 10.1111/mec.16723. 36208022 PMC10092626

[ede70020-bib-0057] Olsson, M. , and T. Madsen . 2001. “Between‐Year Variation in Determinants of Offspring Survival in the Sand Lizard, Lacerta Agilis.” Functional Ecology 15, no. 4: 443–450. http://www.jstor.org/stable/826664.

[ede70020-bib-0058] Olsson, M. , T. Madsen , J. Nordby , E. Wapstra , B. Ujvari , and H. Wittsell . 2003. “Major Histocompatibility Complex and Mate Choice in Sand Lizards.” Proceedings of the Royal Society of London Series B‐Biological Sciences 270: S254–S256. 10.1098/rsbl.2003.0079 PMC1809963 14667398

[ede70020-bib-0059] Olsson, M. , T. Madsen , E. Wapstra , B. Silverin , B. Ujvari , and H. Wittzell . 2005. “MHC, Health, Color, and Reproductive Success in Sand Lizards.” Behavioral Ecology and Sociobiology 58: 1–12.

[ede70020-bib-0060] Olsson, M. , E. Miller , N. Rollings , et al. 2025. “The Effects of Costly Telomere Maintenance on Lifespan: Reproductive Tradeoffs in Sand Lizards.” Evolution; International Journal of Organic Evolution 79: 847–857. 10.1093/evolut/qpae181. 39688874

[ede70020-bib-0061] Olsson, M. , A. Pauliny , E. Wapstra , et al. 2011. “Sexual Differences in Telomere Selection in the Wild.” Molecular Ecology 20, no. 10: 2085–2099. 10.1111/j.1365-294X.2011.05085.x. 21486373

[ede70020-bib-0062] Olsson, M. , and R. Shine . 1996. “Does Reproductive Success Increase With Age or With Size in Species With Indeterminate Growth? A Case Study Using Sand Lizards (Lacerta Agilis).” Oecologia 105: 175–178. 28307079 10.1007/BF00328543

[ede70020-bib-0063] Olsson, M. , and R. Shine . 1997. “The Limits to Reproductive Output: Offspring Size Versus Number in the Sand Lizard (Lacerta Agilis).” American Naturalist 149, no. 1: 179–188. 10.1086/285985.

[ede70020-bib-0064] Olsson, M. , R. Shine , T. Madsen , A. Gullberg , and H. Tegelström . 1996. “Sperm Selection by Females.” Nature 383: 585.

[ede70020-bib-0065] Olsson, M. , E. Wapstra , and C. Friesen . 2018. “Ectothermic Telomeres: It's Time They Came in From the Cold.” Philosophical Transactions of the Royal Society, B: Biological Sciences 373, no. 1741: 20160449. 10.1098/rstb.2016.0449. PMC5784069 29335373

[ede70020-bib-0066] Olsson, M. , E. Wapstra , T. Schwartz , T. Madsen , B. Ujvari , and T. Uller . 2011. “In Hot Pursuit: Fluctuating Mating System AND Sexual Selection in Sand Lizards.” Evolution 65, no. 2: 574–583. 10.1111/j.1558-5646.2010.01152.x. 21044055

[ede70020-bib-0067] Pauliny, A. , E. Miller , N. Rollings , et al. 2018. “Effects of Male Telomeres on Probability of Paternity in Sand Lizards.” Biology Letters 14, no. 8: 20180033. 10.1098/rsbl.2018.0033. 30135115 PMC6127112

[ede70020-bib-0068] Pepke, M. L. , A. K. Niskanen , T. Kvalnes , et al. 2022. “Inbreeding Is Associated With Shorter Early‐Life Telomere Length in a Wild Passerine.” Conservation Genetics 23, no. 3: 639–651. 10.1007/s10592-022-01441-x.

[ede70020-bib-0069] Pfaffl, M. W. 2001. “A New Mathematical Model for Relative Quantification in Real‐Time RT‐PCR.” Nucleic Acids Research 29, no. 9: e45. 10.1093/nar/29.9.e45. 11328886 PMC55695

[ede70020-bib-0070] Plot, V. , F. Criscuolo , S. Zahn , and J.‐Y. Georges . 2012. “Telomeres, Age and Reproduction in a Long‐Lived Reptile.” PLoS One 7, no. 7: e40855. 10.1371/journal.pone.0040855. 22808278 PMC3396605

[ede70020-bib-0071] Reichert, S. , and A. Stier . 2017. “Does Oxidative Stress Shorten Telomeres In Vivo? A Review.” Biology Letters 13, no. 12: 20170463. 10.1098/rsbl.2017.0463. 29212750 PMC5746531

[ede70020-bib-0072] Remot, F. , V. Ronget , H. Froy , et al. 2020. “No Sex Differences in Adult Telomere Length Across Vertebrates: A Meta‐Analysis.” Royal Society Open Science 7, no. 11: 200548. 10.1098/rsos.200548. 33391781 PMC7735339

[ede70020-bib-0073] Ribas‐Maynou, J. , M. Llavanera , Y. Mateo‐Otero , et al. 2022. “Telomere Length in Bovine Sperm Is Related to the Production of Reactive Oxygen Species, but Not to Reproductive Performance.” Theriogenology 189: 290–300. 10.1016/j.theriogenology.2022.06.025. 35816887

[ede70020-bib-0074] Rovatsos, M. , L. Kratochvíl , M. Altmanová , and M. Johnson Pokorná . 2015. “Interstitial Telomeric Motifs in Squamate Reptiles: When the Exceptions Outnumber the Rule.” PLoS One 10, no. 8: e0134985. 10.1371/journal.pone.0134985. 26252002 PMC4529230

[ede70020-bib-0075] Ruijter, J. M. , C. Ramakers , and W. M. H. Hoogaars , et al. 2009. “Amplification Efficiency: Linking Baseline and Bias in the Analysis of Quantitative PCR Data.” Nucleic Acids Research 37, no. 6: e45. 19237396 10.1093/nar/gkp045 PMC2665230

[ede70020-bib-0076] Salmón, P. , and P. Burraco . 2022. “Telomeres and Anthropogenic Disturbances in Wildlife: A Systematic Review and Meta‐Analysis.” Molecular Ecology 31, no. 23: 6018–6039. 10.1111/mec.16370. 35080073 PMC9790527

[ede70020-bib-0077] Sanjak, J. S. , J. Sidorenko , M. R. Robinson , K. R. Thornton , and P. M. Visscher . 2018. “Evidence of Directional and Stabilizing Selection in Contemporary Humans.” Proceedings of the National Academy of Sciences 115, no. 1: 151–156. 10.1073/pnas.1707227114. PMC5776788 29255044

[ede70020-bib-0078] Santiso, R. , M. Tamayo , J. Gosálvez , M. Meseguer , N. Garrido , and J. L. Fernández . 2010. “Swim‐Up Procedure Selects Spermatozoa With Longer Telomere Length.” Mutation Research/Fundamental and Molecular Mechanisms of Mutagenesis 688, no. 1–2: 88–90. 10.1016/j.mrfmmm.2010.03.003. 20226199

[ede70020-bib-0079] Seehuus, S.‐C. , S. Taylor , K. Petersen , and R. M. Aamodt . 2013. “Somatic Maintenance Resources in the Honeybee Worker Fat Body Are Distributed to Withstand the Most Life‐Threatening Challenges at Each Life Stage.” PLoS One 8, no. 8: e69870. 10.1371/journal.pone.0069870. 23940531 PMC3734224

[ede70020-bib-0080] Stearns, S. C. 1976. “Life‐History Tactics: A Review of the Ideas.” Quarterly Review of Biology 51, no. 1: 3–47. 778893 10.1086/409052

[ede70020-bib-0081] Stone, R. C. , K. Horvath , J. D. Kark , E. Susser , S. A. Tishkoff , and A. Aviv . 2016. “Telomere Length and the Cancer–Atherosclerosis Trade‐Off.” PLoS Genetics 12, no. 7: e1006144. 10.1371/journal.pgen.1006144. 27386863 PMC4936693

[ede70020-bib-0082] Sudyka, J. , A. Arct , S. M. Drobniak , L. Gustafsson , and M. Cichoń . 2019. “Birds With High Lifetime Reproductive Success Experience Increased Telomere Loss.” Biology Letters 15, no. 1: 20180637. 10.1098/rsbl.2018.0637. 30958221 PMC6371901

[ede70020-bib-0083] Tobler, M. , D. Gómez‐Blanco , A. Hegemann , et al. 2022. “Telomeres in Ecology and Evolution: A Review and Classification of Hypotheses.” Molecular Ecology 31, no. 23: 5946–5965. 10.1111/mec.16308. 34865259

[ede70020-bib-0084] Tuomi, J. M. , F. Voorbraak , D. L. Jones , and J. M. Ruijter . 2010. “Bias in the Cq Value Observed With Hydrolysis Probe Based Quantitative PCR Can Be Corrected With the Estimated PCR Efficiency Value.” Methods 50, no. 4: 313–322. 20138998 10.1016/j.ymeth.2010.02.003

[ede70020-bib-0085] Ujvari, B. , P. A. Biro , J. E. Charters , et al. 2017. “Curvilinear Telomere Length Dynamics in a Squamate Reptile.” Functional Ecology 31, no. 3: 753–759. 10.1111/1365-2435.12764.

[ede70020-bib-0086] Ujvari, B. , and T. Madsen . 2009. “Short Telomeres in Hatchling Snakes: Erythrocyte Telomere Dynamics and Longevity in Tropical Pythons.” PLoS One 4, no. 10: e7493. 10.1371/Journal.Pone.0007493. 19834611 PMC2759514

[ede70020-bib-0087] Ujvari, B. , N. Raven , T. Madsen , et al. 2022. “Molecular Ecology Telomeres, the Loop Tying Cancer to Organismal Life‐Histories.” Molecular Ecology 31, no. n/a: 6273–6285. 10.1111/mec.16488. 35510763 PMC9790343

[ede70020-bib-0088] Verhulst, S. , A. Aviv , A. Benetos , G. S. Berenson , and J. D. Kark . 2013. “Do Leukocyte Telomere Length Dynamics Depend on Baseline Telomere Length? An Analysis That Corrects for ‘Regression to the Mean.” European Journal of Epidemiology 28, no. 11: 859–866. 10.1007/s10654-013-9845-4. 23990212

[ede70020-bib-0089] Vertecchi, E. , A. Rizzo , and E. Salvati . 2022. “Telomere Targeting Approaches in Cancer: Beyond Length Maintenance.” International Journal of Molecular Sciences 23, no. 7: 3784. 10.3390/ijms23073784. 35409143 PMC8998427

[ede70020-bib-0090] Voituron, Y. , O. Guillaume , A. Dumet , S. Zahn , and F. Criscuolo . 2023. “Temperature‐Independent Telomere Lengthening With Age in the Long‐Lived Human Fish (*Proteus anguinus*).” Proceedings of the Royal Society B: Biological Sciences 290, no. 1998: 20230503. 10.1098/rspb.2023.0503. PMC10154926 37132239

[ede70020-bib-0091] Wilbourn, R. V. , J. P. Moatt , H. Froy , C. A. Walling , D. H. Nussey , and J. J. Boonekamp . 2018. “The Relationship Between Telomere Length and Mortality Risk in Non‐Model Vertebrate Systems: A Meta‐Analysis.” Philosophical Transactions of the Royal Society, B: Biological Sciences 373, no. 1741: 20160447. 10.1098/rstb.2016.0447. PMC5784067 29335371

[ede70020-bib-0092] Xiong, Y. , R. Rozzi , Y. Zhang , et al. 2024. “Convergent Evolution Toward a Slow Pace of Life Predisposes Insular Endotherms to Anthropogenic Extinctions.” Science Advances 10, no. 28: adm8240. 10.1126/sciadv.adm8240. PMC11244536 38996028

[ede70020-bib-0093] Zade, N. H. , and E. Khattar . 2023. “POT1 Mutations Cause Differential Effects on Telomere Length Leading to Opposing Disease Phenotypes.” Journal of Cellular Physiology 238, no. 6: 1237–1255. 10.1002/jcp.31034. 37183325

[ede70020-bib-0094] Zakharov, V. M. 1989. “Future Prospects for Population Phenogenetics.” Soviet Scientific Reviews Section F, Physiology and General Biology Reviews 4: 1–79.

